# Radiomics analysis of uterine tumors in 18F-fluorodeoxyglucose positron emission tomography for prediction of lymph node metastases in endometrial carcinoma

**DOI:** 10.55730/1300-0144.5371

**Published:** 2022-02-27

**Authors:** Çiğdem SOYDAL, Bulut VARLI, Mine ARAZ, Batuhan BAKIRARAR, Salih TAŞKIN, Uğur Fırat ORTAÇ

**Affiliations:** 1 Department of Nuclear Medicine, Faculty of Medicine, Ankara University, Ankara, Turkey; 2 Department of Gynecological Oncology, Faculty of Medicine, Ankara University, Ankara, Turkey; 3 Department of Biostatistics, Faculty of Medicine, Ankara University, Ankara, Turkey

**Keywords:** Endometrial carcinoma, lymph node metastasis, ^18^F-FDG PET, CT, radiomics

## Abstract

**Background/aim:**

In this single-center study, we aimed to analyze texture features of primary uterine lesions on 18F-FDG PET/CT to predict lymph node metastases.

**Material and methods:**

Totally, 157 (mean age: 62 ± 10.2 years) patients were included in the analysis. Histopathological examination results were considered as the standard reference for nodal involvement. On ^18^F-FDG PET/CT images, only the primary tumor was segmented. SUVmax, SUVmean, SUVpeak, MTV, and TLG of primary uterine lesions were calculated for analyses. For texture analysis first, second, and higher-order texture features were calculated.

**Results:**

Mean diameter of primary uterine lesions was calculated as 35± 18.1 mm. Lymph node metastases were detected in 19% of patients in histopathological examination of surgical materials. While 26 patients had pelvic lymph node metastases, 19 patients had additional paraaortic lymph node metastases. On radiomics analysis for 20 features, a significant difference was found between patients with and without lymph node metastasis. With using data mining methods GLZLM ZLNU, Entropy_GLCM_, Entropy_histo_, GLRLM LRHGE, GLZLM HGZE, GLZLM SZHGE, GLRLM HGRE, GLRLM SRHGE were found significant radiomics features to predict lymph node metastasis with a diagnostic accuracy of 0.8.

**Conclusion:**

The radiomics analysis of intratumoral heterogeneity is a promising method for improving triage of the patients for lymph node dissection in endometrial carcinoma.

## 1. Introduction

Endometrial carcinoma is the most common gynecological cancer worldwide [1]. Lymph nodes are the most common extrauterine spread site with the rate of 20% and the presence of lymph node metastasis is a prognostic factor for endometrial carcinoma patients [2,3]. Although surgery including bilateral pelvic and paraaortic lymphadenectomy is gold standard for nodal staging, the surgical management of the nodal disease is still controversial [4,5]. Randomized trials showed that pelvic lymphadenectomy had no impact on survival in early-stage endometrial carcinoma patients [6,7]. Considering the low rate of lymph node metastases, determination of lymph node status preoperatively would be beneficial for patient management to avoid surgery-related complications. In the last decade, sentinel lymph node (SLN) mapping has become a feasible option with high diagnostic accuracy in the management of clinically early-stage endometrial cancer [8]. However, the SLN algorithm suggests resection of only the mapped nodes without systematic lymphadenectomy even if the metastasis exists in the SLNs. Moreover, the status of SLNs for metastasis will be reported in the postoperative period and patients with SLN metastasis may have a need of a second surgery for systematic lymphadenectomy. Another limitation of SLN mapping is that it primarily focuses on pelvic lymph nodes and evaluation of the paraaortic area is not a standard. Thus, with this approach, isolated paraaortic metastases can be under-diagnosed [9,10].

With its high positive predictive value, ^18^F-FDG PET/CT is suggested as the best technique for endometrial carcinoma nodal staging, particularly in high-risk patients [11]. However, due to its low spatial resolution, metastatic lymph nodes smaller than 5 mm result in false-negative results [12]. Parameters reflecting the amount of ^18^F-FDG uptake in the lymph node or in primary uterine lesion have been studied. These were standardized uptake value (SUV) based parameters such as SUVmax, SUVpeak, SUVmean as well as metabolic tumor volume (MTV) and total lesion glycolysis (TLG) [12]. However, tumor ^18^F-FDG uptake shows the uneven spatial distribution, at least partly due to underlying biological tumor conditions such as metabolism, hypoxia, necrosis, and cellular proliferation, which is called intratumoral heterogeneity [12]. One of the most highlighted methods to quantify intratumoral heterogeneity from images is texture analysis. Because intratumoral heterogeneity is related to tumor aggressiveness, treatment response, and prognosis, the relationship between texture features of primary tumor focus, and lymph node metastases has been a subject of interest. Recently, a few studies have focused on the value of radiomics features of primary uterine tumors on PET images to improve sensitivity in the detection of lymph node metastases in patients with endometrial carcinoma [13,14]. In this single-center study, we aimed to analyze texture features of primary uterine lesions on 18F-FDG PET/CT to predict lymph node metastases.

## 2. Material and methods

### 2.1. Patient population

In this study, ^18^F-FDG PET/CT images of 191 patients who underwent surgical treatment for endometrial carcinoma were evaluated retrospectively. The study was approved by the local ethical committee (approval number: İ1-40-21). Nineteen patients were excluded because their primary tumors were non ^18^F-FDG avid. Additional 15 patients, with a tumor smaller than 64 voxels were excluded from the analysis. Finally, images of 157 patients were included in the analysis. All the included patients had undergone total hysterectomy, bilateral salpingo-oophorectomy, and at least bilateral pelvic lymphadenectomy. Histopathological examination results were considered as the standard reference for nodal involvement.

### 2.2. ^18^F-FDG PET/CT protocol and tumor segmentation

All the patients underwent ^18^FDG PET/CT for evaluation of the disease stage. Informed consent was obtained from all patients before imaging. PET/CT images were acquired with two PET/CT scanners, a GE Discovery ST and Discovery 710 (GE Medical Systems, Milwaukee, USA). Patients fasted at least 6 h before imaging and blood glucose levels were checked. Those with a blood glucose level above 150 mg/dL did not undergo scanning. Oral contrast was given to all patients. Images from the vertex to the proximal femur were obtained in the supine position. Whole-body ^18^F-FDG PET/CT imaging was performed approximately 1 h after intravenous injection of 296–370 MBq ^18^F-FDG. During the waiting period, patients rested in a quiet room without taking any muscle relaxants. PET images were acquired for 4 min per bed position. Emission PET images were reconstructed with noncontrast CT images. CT images were also obtained with a standardized protocol of 140 kV, 70 mA, tube rotation time of 0.5 s per rotation, a pitch of 6, and a slice thickness of 5 mm. Patients were allowed to breathe normally during the procedure. Attenuation-corrected PET/CT fusion images were reviewed in three planes (transaxial, coronal, and sagittal) on an AW VolumeShare 7 (GE Medical Systems, Milwaukee, USA) workstation. A board-certified nuclear medicine physician with more than 10 years’ experience in PET/CT, segmented primary uterine lesions. ^18^F-FDG PET/CT images were evaluated with a semiautomatic approach. The volume of interest (VOI) of the uterine lesion was defined on PET images with a threshold of 40% of the maximum standardized uptake value (SUVmax) using commercial software (PET VCAR; GE Healthcare). Only the primary tumor was segmented. SUVmax, SUVmean, SUVpeak, MTV, and TLG of primary uterine lesions were calculated for analyses.

### 2.3. Texture feature extraction

**Figure 1:**
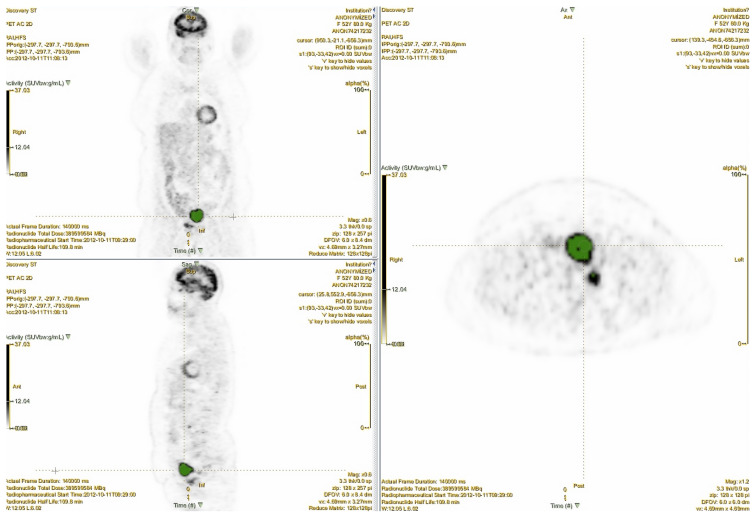
An example for tumor delineation for texture analysis.

Texture features (i.e. first-, second-, and higher-order imaging parameters) were extracted using dedicated software for radiomics (LIFEx) (https://www.lifexsoft.org/index.php). For technical reasons, second and higher-order imaging parameters were extracted only for lesions greater than 64 voxels. Details of calculated first, second, and higher-order texture features are given in Table 1. An example of tumor delineation for texture analysis is given in Figure 1.

### 2.4. Statistical analysis

WEKA 3.7 and SPSS 11.5 programs were used to evaluate the data. Descriptives were presented as mean ± standard deviation and median (minimum-maximum) for quantitative variables and number of patients (percent) for qualitative variables. In order to investigate whether there is a statistically significant difference between the qualitative variables with lymph node metastasis positive and negative groups. Mann-Whitney U test was used since the normal distribution assumptions were not met. The statistical significance level was taken as 0.05. Classification methods of Support Vector Machine, Hoeffding Tree, J48, and Multilayer Perceptron were used in the WEKA program. The data set was evaluated using the 10-fold cross-validation test option. Accuracy, F-Measure, Precision, Recall, and Precision-Recall Curve (PRC Area) were used as data mining performance criteria of textural features of primary uterine tumors to predict lymph node metastasis.

## 3. Results

### 3.1. Patients

Images of 157 (mean age: 62 ± 10.2 years) women who underwent an ^18^F-FDG PET/CT scan with the diagnosis of endometrial carcinoma between March 2012 and July 2019 were analyzed. Details of the study population are presented in Table 2. The mean diameter of primary uterine lesions was calculated as 35 ± 18.1 mm. Lymph node metastases were detected in 30 (19%) of patients in histopathological examination of surgical materials. While 26 patients had pelvic lymph node metastases, 19 patients had paraaortic lymph node metastases.

### 3.2. Texture analysis of ^18^F-FDG PET images and data mining

Descriptive data of texture parameters for lymph node metastasis positive and negative patient groups are provided in Table 3. On radiomics analysis for 20 features, a significant difference was found between patients with and without lymph node metastasis.

**Figure 2:**
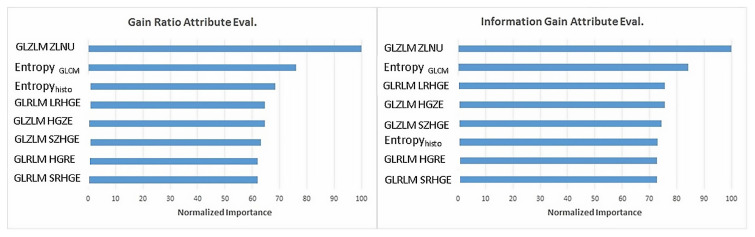
Variable importance for metastases.

Information Gain Attribute Eval and Gain Ratio Attribute Eval methods in WEKA were used because there were too many variables in the data set. With using these methods, the importance of the variables and the values it added to the data set were examined. The variables, which were determined to be insignificant by two methods and considered to be unimportant as clinical information, were excluded from the data set. A total of 9 variables (8 independent variables and 1 dependent variable) remained finally. These variables were GLZLM ZLNU, Entropy_GLCM_, Entropy_histo_, GLRLM LRHGE, GLZLM HGZE, GLZLM SZHGE, GLRLM HGRE, GLRLM, SRHGE, and lymph node metastases. Percentages of variable importance according to dependent variable lymph node metastases are given in Figure 2.

Looking at the data mining results in Table 4, the Support Vector Machine, Hoeffding Tree, and J48 gave similar results according to Accuracy and F-measure criteria, which are the most accepted performance criteria. Multilayer perceptron was found to be the worst serving method. The diagnostic accuracy of texture parameters was calculated at about 0.8 for the prediction of lymph node metastasis.

## 4. Discussion

Due to the limited spatial resolution of ^18^F-FDG PET, metastatic lymph nodes smaller than 5 mm cannot be diagnosed accurately and this can cause false-negative findings [11]. In this study, we aimed to investigate the efficacy of radiomics analysis of the primary uterine lesion to improve the sensitivity of ^18^F-FDG PET in detecting nodal metastases. We analyzed standard imaging features like SUV, MTV, and TLG, together with the first, second, and higher-order texture features.

The concept of radiomics is defined as the high-throughput extraction of a large number of features from medical images [15,16]. It is assumed that genomic and proteomic cancer patterns are expressed in image-based features and with optimal analysis of medical images such as texture analysis, cancer properties can be quantified [12,16]. Texture analysis has long been applied in CT and magnetic resonance imaging (MRI), but it has been adapted to PET imaging very recently [14]. As the most widely used PET feature; SUVmax of primary uterine lesions, is considered as an important indicator reflecting tumor aggressiveness, such as myometrial invasion or tumor grade. However, it was not significantly correlated with lymph node status according to previous studies [17,18]. Contrarily to SUVmax other semiquantitative parameters, MTV and TLG seem to have a predictive role for lymph node status. Endometrial tumors with higher MTV and TLG have a higher tendency to spread to lymph nodes than without [19].

In our analyses, descriptive parameters of SUVpeak, MTV, and TLG of patients with and without lymph node metastasis were found significantly different. However, they did not remain significant in the data mining methods. In the data mining methods, one first, one second, and six higher-order texture parameters were found significant to predict lymph node metastases. GLZLM ZLNU showed the best performance for nodal staging in data mining. It is a higher-order texture feature and is described as the length of the homogeneous zones in the gray level zone length matrix. It has been reported as the best texture parameter for discrimination of squamous cell carcinoma and adenocarcinoma of the lung [20]. Similarly, patients with estrogen negative breast tumors were reported higher GLZLM ZLNU than positive ones [21]. Moreover, Wang et al. published prognostic value of functional parameters of ^18^F-FDG PET/CT in patients with renal/adrenal lymphoma. They have reported that the tumor stage, Entropy _GLCM_, GLZLM GLNU, and GLZLM ZLNU values were significant predictors of overall survival [22]. Entropy _GLCM_ which is described as the randomness of grey-level voxel pairs was the second most significant texture feature in our series.

When we take a look at the PET radiomics studies in endometrial carcinoma patients, data is limited in the literature [13,14]. Bernardi et al. reported their data for texture analysis of ^18^F-FDG PET images of endometrial carcinoma patients. They found that the zone percentage of the grey level size zone matrix, (GLSZM ZP) was able to predict LN metastases better than any other feature. Also, they reported that the combination of visual detection and GLSZM ZP values increased the sensitivity of lymph node metastasis detection [13]. In another study, Crivellaro et al. reported the results of the combination of PET/CT, radiomics, and sentinel lymph node mapping for nodal staging of endometrial cancer patients. They found a significant association between the presence of lymph node metastases and 64 features on radiomics analysis. Volume-density was the most predictive feature. They concluded that PET radiomics features of the primary tumor seemed promising for the prediction of nodal metastases, which were not detected by visual analysis [14]. In endometrial carcinoma patients, different radiomics features have been reported as the best predictor for lymph node metastases. That might be related to the limited number of included patients and the different distribution of risk groups between studies. In our study, differently from previous ones, we used cross-validation method for data mining. Cross-validation is the recommended method for analyzing all data with more precise metrics [23]. Although different texture features showed significant distribution among patients with and without lymphatic metastases, results of our analysis and previously reported ones support the idea that radiomics analysis of PET images of primary uterine lesions is a promising method to define lymph node metastasis. Standardized, randomized studies with large patient populations would help validation of texture features to predict lymph node metastasis of endometrial carcinoma.

In routine clinical practice, lymphadenectomy decision is given according to risk factors-grade, myometrial invasion and tumor type- provided by preoperative endometrial biopsy or intraoperative frozen section examination. However, unnecessary lymphadenectomy is still possible for many patients despite the use of these methods. Instead of this approach, the SLN algorithm seems to be a more convenient method, but low volume metastases are also diagnosed by the SLN ultrastaging procedure, and the sensitivity of ^18^F-FDG PET CT in the diagnosis of lymphatic metastases decreases. With the combined use of radiomics and SLN mapping [14], patients who are candidates for SLN mapping and ultrastaging can be determined, even for low-volume disease, and patients at risk for lymphatic metastasis can be more accurately identified. We planned to investigate the diagnostic accuracy of a combination of SLN mapping and radiomics in another study.

This study is one of the very few studies evaluating radiomics in endometrial cancer, and our results may guide future studies. However, the retrospective design and the small number of patients were the major limitations of our study. Although we have not correlated oncologic outcomes with radiomics analysis, future studies may show a correlation of these two features and this knowledge could help clinicians in planning patient-based treatment and follow-up protocols. In this study, we analyzed data of two different PET/CT scanners. It should be kept in mind that accurate results cannot be achieved in the data of two different PET/CT scanners, even if they were performed using the same protocol. Since this may cause heterogeneity in the images and the results obtained. Another limitation of the study is that the radiomics analysis results in combination with SLN mapping are missing. Lastly, dealing with high data input made results technically complex. As the studies come across, several models would be created, and this complex situation could then be overcome.

## 5. Conclusion

The radiomics analysis of intratumoral heterogeneity is a promising method for improving triage of the patients for lymph node dissection in endometrial carcinoma. However, multicenter studies with larger patient populations are needed for validation.

## Figures and Tables

**Table 1:** List of the studied radiomics features.

The first-order	The higher-order
SUVmax	GLRLM SRE
SUVmean	GLRLM LRE
SUVpeak	GLRLM LGRE
MTV	GLRLM HGRE
TLG	GLRLM SRLGE
Skewness	GLRLM SRHGE
Kurtosis	GLRLM LRLGE
Entropy_histo_	GLRLM LRHGE
Energy	GLRLM GLNU
SHAPE Sphericity	GLRLM RLNU
SHAPE Compacity	GLRLM RP
	GLZLM SZE
**The second-order**	GLZLM LZE
Homogenity _GLCM_	GLZLM LGZE
Energy _GLCM_	GLZLM HGZE
Contrast _GLCM_	GLZLM SZLGE
Correlation _GLCM_	GLZLM SZHGE
Entropy _GLCM_	GLZLM LZLGE
Dissimilarity _GLCM_	GLZLM LZHGE
	GLZLM GLNU
	GLZLM ZLNU
	GLZLM ZP
	Coarseness _NGLDM_
	Contrast _NGLDM_
	Busyness _NGLDM_

*SUV* standardized uptake value, *MTV* metabolic tumor volume, *TLG* total lesion glycolysis, *GLCM* gray-level cooccurence matrix, *GLRLM* gray-level run-length matrix, *SRE* short-run emphasis, *LRE* long-run emphasis, *LGRE* low gray-level run emphasis, *HGRE* high gray-level run emphasis, *SRLGE* short-run low gray-level emphasis, *SRHGE* short-run high gray-level emphasis, *LRLGE* long-run low gray-level emphasis, *LRHGE* long-run high gray-level emphasis, *GLNU* gray-level nonuniformity, *RP* run percentage, *GLZLM* gray level zone length matrix, *SZE* short-zone emphasis, *LZE* long-zone emphasis, *LGZE* low gray-level zone emphasis, *HGZE* high gray-level zone emphasis, *SZLGE* short-zone low gray-level emphasis, *SZHGE* short-zone high gray-level emphasis, *LZLGE* long-zone low gray-level emphasis, *LZHGE* long-zone high gray-level emphasis, *ZLNU* zone length nonuniformity, *ZP* zone percentage, *NGLDM* neighborhood grey-level different matrix.

**Table 2:** Characteristics of the patient population.

Parameter	
*Age (mean ± SD, years)*	62.0 ± 10.2
*Menopausal state (n,%)*	
Premenopausal	25 (15.9%)
Postmenopausal	132 (84.1%)
*Grade (n,%)*	
G1	44 (28.0%)
G2	56 (35.6%)
G3	57 (36.4%)
*Histology (n, %)*	
Endometrioid	124 (79.0%)
Clear cell/serous/mucinous/mixed	21 (13.4%)
Malignant mesodermal tumor	12 (7.6%)
*Myometrial invasion (n %)*	
None	11 (7.0%)
<50%	74 (47.1%)
>50%	72 (45.9%)
*FIGO Stage (n, %)*	
I	112 (71.3%)
II	12 (7.6%)
III	26 (16.6%)
IV	7 (4.5%)
*Lymph node metastases (n,%)*	
Yes	30 (19.1%)
No	127 (80.9%)

*FIGO:* Federation of Gynecology and Obstetrics

**Table 3:** Descriptives for lymph node metastases.

Variables	Lymph node metastasis				
	No (n = 127)		Yes (n = 30)		
	Mean ± SD	Median	Mean ± SD	Median	p value
		(min–max)		(min–max)	
SUVpeak	8.88 ± 7.81	9^th^ June	12.83 ± 7.35	15.13	0.008
		(0.00–30.28)		(0.00–27.67)	
TLG	115.81 ± 180.55	45.55	300.17 ± 418.73	156.97	<0.001
		(5.13–1227.41)		(9.21–2209.97)	
Entropy_histo_	1.19 ± 0.21	January 22	1.28 ± 0.25	January 36	0.002
		(0.00–1.58)		(0.60–1.57)	
Energy	0.09 ± 0.10	0.07	0.09 ± 0.09	0.05	0.007
		(0.03–1.00)		(0.03–0.47)	
MTV	11.55 ± 13.83	June 18	25.11 ± 29.59	14.29	<0.001
		(0.52–85.00)		(2.01–150.60)	
Energy _GLCM_	0.02 ± 0.03	0.01	0.02 ± 0.06	0.01	0.037
		(0.00–0.34)		(0.00–0.34)	
Contrast _GLCM_	57.29 ± 52.40	47.50	73.94 ± 43.28	66.39	0.032
		(0.00–228.46)		(0.00–174.36)	
Correlation _GLCM_	0.17 ± 0.15	0.16	0.28 ± 0.18	0.24	0.004
		(−0.10–0.58)		(0.00–0.63)	
Entropy _GLCM_	1.61 ± 0.84	January 84	2.10 ± 0.69	February 24	<0.001
		(0.00–2.75)		(0.00–2.82)	
Dissimilarity _GLCM_	4.98 ± 3.20	May 43	6.26 ± 2.52	June 29	0.036
		(0.00–12.14)		(0.00–10.72)	
GLRLM HGRE	849.27 ± 891.45	550.55	1325.28 ± 825.50	1337.62	0.001
		(0.00–3446.93)		(0.00–3584.94)	
GLRLM SRHGE	803.31 ± 814.99	536.24	1245.22 ± 707.57	1294.08	0.002
		(0.00–3044.55)		(0.00–2630.25)	
GLRLM LRHGE	1332.78 ± 3300.98	609.35	2118.29 ± 3716.78	1472.90	0.001
		(0.00–34583.96)		(0.00–21,168.33)	
GLRLM GLNU	15.45 ± 29.84	August 25	21.64 ± 21.04	14^th^ June	0.014
		(0.00–279.51)		(0.00–84.61)	
GLRLM RLNU	174.91 ± 229.61	108.71	376.92 ± 390.69	242.75	0.001
		(0.00–1453.19)		(0.00–1778.01)	
GLZLM HGZE	822.53 ± 816.52	564.47	1294.76 ± 751.74	1339.45	0.001
		(0.00–3153.00)		(0.00–3164.98)	
GLZLM SZHGE	630.12 ± 660.65	382.74	984.46 ± 627.32	980.51	0.002
		(0.00–2444.10)		(0.00–2828.95)	
GLZLM LZHGE	102,276.19 ± 903,259.46	2686.92	116,072.17 ± 578,882.13	6288.71	0.005
		(0.00–10,085,856.18)		(0.00–3,179,985.77)	
GLZLM GLNU	6.17 ± 6.96	4^th^ July	10.84 ± 9.47	August 34	0.002
		(0.00–36.85)		(0.00–43.27)	
GLZLM ZLNU	54.79 ± 68.57	30.25	115.81 ± 111.29	95.10	<0.001
		(0.00–361.54)		(0.00–566.81)	

*SUV* standardized uptake value, *MTV* metabolic tumor volume, *TLG* total lesion glycolysis, *GLCM* gray-level cooccurence matrix, *GLRLM* gray-level run-length matrix, *HGRE* high gray-level run emphasis, *SRHGE* short-run high gray-level emphasis, *LRHGE* long-run high gray-level emphasis, *GLNU* gray-level nonuniformity, *HGZE* high gray-level zone emphasis, *SZHGE* short-zone high gray-level emphasis, *LZHGE* long-zone high gray-level emphasis, *ZLNU* zone length nonuniformity.

**Table 4:** Performance comparison of data mining methods.

Methods	F-Measure	Precision	Recall	PRC Area
**Support vector machine**	0.723	0.655	0.808	0.691
**Hoeffding Tree**	0.723	0.655	0.808	0.688
**J48**	0.731	0.720	0.803	0.701
**Multilayer perceptron**	0.736	0.716	0.771	0.745
